# Memory markers in the continuum of the Alzheimer’s clinical syndrome

**DOI:** 10.1186/s13195-022-01082-9

**Published:** 2022-09-30

**Authors:** Mario A. Parra, Clara Calia, Vivek Pattan, Sergio Della Sala

**Affiliations:** 1 School of Psychological Sciences and Health, University of Strathclyde, Graham Hills Building, 40 George Street, Glasgow, G1 1QE UK; 2 School of Health in Social Science, University of Edinburgh, Edinburgh, UK; 3 NHS Forth Valley, Stirling Community Hospital, Stirling, UK; 4 Human Cognitive Neuroscience, Psychology Department, University of Edinburgh, Edinburgh, UK

**Keywords:** Visual Short-Term Memory Binding, Alzheimer’s disease, Neuropsychological assessment, Early detection

## Abstract

**Background:**

The individual and complementary value of the Visual Short-Term Memory Binding Test (VSTMBT) and the Free and Cued Selective Reminding Test (FCSRT) as markers to trace the AD continuum was investigated. It was hypothesised that the VSTMBT would be an early indicator while the FCSRT would inform on imminent progression.

**Methods:**

Healthy older adults (*n*=70) and patients with mild cognitive impairment (MCI) (*n*=80) were recruited and followed up between 2012 and 2017. Participants with at least two assessment points entered the study. Using baseline and follow-up assessments four groups were defined: Older adults who were healthy (HOA), with very mild cognitive but not functional impairment (eMCI), and with MCI who did and did not convert to dementia (MCI converters and non-converters).

**Results:**

Only the VSTMBT predicted group membership in the very early stages (HOA vs eMCI). As the disease progressed, the FCSRT became a strong predictor excluding the VSTMB from the models. Their complementary value was high during the mid-prodromal stages and decreased in stages closer to dementia.

**Discussion:**

The study supports the notion that neuropsychological assessment for AD needs to abandon the notion of one-size-fits-all. A memory toolkit for AD needs to consider tools that are early indicators and tools that suggest imminent progression. The VSTMBT and the FSCRT are such tools.

**Supplementary Information:**

The online version contains supplementary material available at 10.1186/s13195-022-01082-9.

## Background

Alzheimer’s disease (AD) has been defined as a continuum of clinical and pathological events from normal ageing to dementia. Accordingly, the disease has been reconceptualised [1–4] and new diagnostic frameworks relying on biomarkers have been introduced [5, 6]. The motivation behind these biomarkers-based frameworks has been the limitations that available neuropsychological tests have demonstrated in detecting the pre-dementia stages of such a continuum [3, 7, 8]. In the 10 years since Sperling et al. [3] suggested the relevance of the long preclinical stages of AD, the emphasis has shifted from the study of mild cognitive impairment (MCI) and progression to dementia to the study of incident cognitive impairment linked to risk of dementia. Recent evidence following such recommendations suggests that neuropsychological measures may provide meaningful signals informing on the different stages of preclinical AD in still cognitively intact older adults [9]. Memory is the cognitive function earliest and most dramatically impacted by the typical forms of AD [8–11]. However, accrued evidence suggests that neuropsychological assessment needs a paradigm shift if we are to enhance its sensitivity and specificity for the preclinical stages of the disease [8, 12–14].

**Fig. 1:**
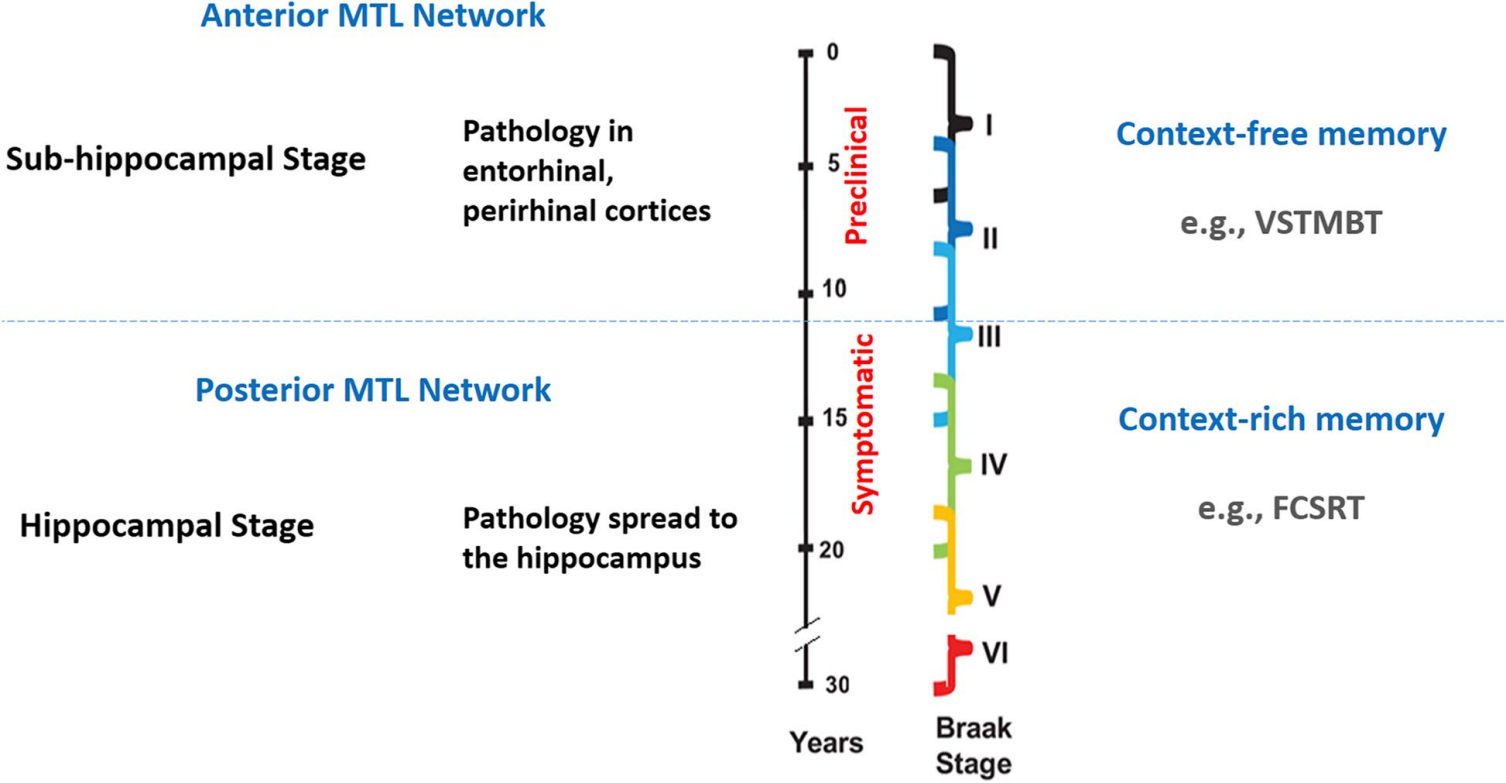
Diagram based on Didic et al.’s [12] model. It maps the two tests investigated here (VSTMBT and FCSRT) onto the stages of AD. It follows the rationale of the model to suggest when along the continuum, these tests could become most informative. Stage III of Braak [20, 21] corresponds to the onset of MCI (spread of pathology from the anterior medial temporal lobe (MTL) network to the posterior network). Based on the above hypothesis and that proposed by Parra [22], we predicted that the VSTMBT, as a context-free memory test, would inform on the risk of progressing from normal to pathological ageing (e.g. early MCI or objectively defined subtle cognitive decline [23]) while the FCSRT, as a context-rich memory test, would inform on the risk of progressing to AD dementia

Recent recommendations by the Joint Program for Neurodegenerative Diseases Working Group [10] fit well with the hypothetical model of memory decline in AD originally proposed by Didic et al. [12] (see Fig. 1). The Working Group recommended two memory tests that have recently proved useful in the assessment of preclinical AD: the Visual Short-Term Memory Binding Test (VSTMBT [15];) and the Free and Cued Selective Reminding Test (FCSRT [16];). Both tests assess the ability to integrate information in memory. However, they tap into different memory functions. Visual Short-Term Memory Binding (VSTMB) refers to our ability to integrate objects’ features into unified representations to form and temporarily hold new identities in memory [17–19]. Typically, the VSTMBT assesses this ability by asking people to recognise changes in coloured shapes or objects occurring between two consecutive displays (i.e. study and test display of change detection tasks). To detect such changes accurately, participants do not need contextual information, rather they need to judge if the newly presented object (test display) is the same as previously presented or different.

The FCSRT, as well as other tests that follow the selective reminding paradigm, such as the memory capacity test (MCT, [24–26]), rely on contextual information to support both encoding and retrieval. The assumption of these tests is that if the contextual cues presented during the encoding (i.e. semantic categories) match those available during recall, they would assist the retrieval of exemplar memories linked to such categories (i.e. the encoding specificity principle [25]). Binding items (i.e. exemplars) to their context (i.e. semantic categories) effectively, should aid memory performance in the context of tests such as the FCSRT and MCT.

The underlying construct of both these two tests seems to be *memory binding*. However, they appear to tax two very different binding functions. The VSTMBT assesses a form of conjunctive binding responsible for holding integrated features within object representations, whereas the FCSRT assesses a form or relational binding that supports the retention of associative memories. We briefly illustrate what Didic et al.’s [12] model implicates about these forms of binding in the AD continuum next in Fig. 1.

Traditionally, studies that have investigated the predictive value of neuropsychological tests to anticipate who among those at risk of AD will eventually develop dementia have conducted retrospective analyses comparing baseline performance of patients in predementia stages (e.g. MCI) who did and did not convert to dementia in the follow-up period. Given the evidence summarised above, such prediction models are most likely to be informative for neuropsychological tests sensitive to advanced stages of the AD continuum (i.e. limbic stage). A function that has started to decline years before people become aware of any cognitive impairments or develop initial symptoms of dementia, may have declined dramatically by the time they reach the MCI stage. At this point in time, separating MCI patients who will and will not develop dementia in 2 or 3 years may be problematic for such tests but may still be possible for tests that assess functions sensitive to the limbic stages, that exhibit a less steep decline (see for example [27, 28]), or that are compensated by protective factors, such as cognitive reserve [29, 30]. Parra et al. [31] recently suggested that the VSTMBT may need to be titrated to the targeted population (e.g. preclinical or prodromal) by adjusting memory load (i.e. 2 or 3 items) in order to achieve best classification power. Similarly, it has been suggested that future studies using biomarkers will need to rely on adjusted normative data in order to ascertain who the true control participants are [32]. In a conference paper, Parra et al. [33] reported that older adults who are completely asymptomatic but show poor VSTMB abilities have significantly more accumulation of amyloid deposits in the brain than those whose binding abilities are spared.

We posit that both the VSTMBT and the FCRST will be able to correctly classify most of the older adults at different stages of the continuum of the AD clinical syndrome, but that their discrimination power would differ depending on where people are in such a continuum. We predict that the VSTMBT will be able to discriminate between older adults who are asymptomatic and those who are in the very early stages of cognitive decline more effectively than the FCSRT (H1). On the contrary, the FCSRT will discriminate more accurately than the VSTMBT between older adults with MCI who converted and who did not convert to dementia in follow-up assessments (H2).

## Methods

### Participants

We recruited participants self-reporting as being healthy who were either members of the Psychology Volunteer Panel at the University of Edinburgh or relatives of patients with dementia from the Scottish Dementia Clinical Research Network interest register (SDCRN, currently Neuroprogressive and Dementia Network - NDN) who volunteered for the study. We also received referrals from old age psychiatrists based at the NHS Lothian and NHS Forth Valley who regularly see older adults complaining about their cognitive abilities. Recruitment and follow-up assessments ran between 2012 and 2017. To be eligible for the study, participants had to be over 55 years old and native English speakers. MCI patients had to have an available relative or a caregiver and demonstrate the capacity to consent to the study. All the participants needed to be free from any neurological or psychiatric disease that would interfere with their cognitive functions, and had normal or corrected to normal vision. Participants with scores greater than 4 in the Hachinski Ischemia Scale [34] and 5 in the brief Geriatric Depression Scale (GDS, [35]) were not included in the final sample. Also, participants who met the criteria for AD dementia at baseline were not eligible. All participants were provided with an Information Sheet describing the longitudinal nature of the study and the assessments involved. They were told that their cognitive and functional abilities would be assessed, that it was possible to detect impairments of which they were not aware, that should this happen, their GPs would be contacted with their consent. After they read the PIS, they signed a consent form prior to participating in the study. The study was approved by the NHS Multi-Site Research Ethics Committee (reference number 06/MRE07/40) and was given approval by local NHS R&D offices. In addition to the above criteria, only participants who had completed at least two assessment points including baseline were entered into the analyses here reported. The final sample consisted of 150 participants. Of these, 70 self-reported as healthy and 80 were referred by consultant old age psychiatrists as patients meeting the criteria for MCI.

### Sample design and rationale

Power calculation was performed which incorporated (1) pilot data obtained from 23 MCI patients and 30 controls as well as from 14 mild AD patients all assessed with the VSTMBT proposed here. In addition, a wide search of the literature was performed to obtain three main variables: (1) average follow-up period within which changes could be observed using sensitive cognitive tasks (3 years, [36]), (2) MCI to AD conversion rate (median = per annum 12%, 37.65% for a 3-year study), and (3) attrition (14% for a 3-year follow-up study). The results showed that for a desired power of 80%, a medium effect size (Cohen *d* = 0.5) and alpha set at 0.05, 80 MCI patients and 40 controls at baseline would allow us to reach the study end-point with a number of converters which permits reliable comparisons (≥ 20).

Baseline data were used to define groups by applying classical MCI criteria [37–39]. We relied on tests for which valid norms had been previously published (see “Neuropsychological assessment” section). Participants were allocated to the Healthy Older Adults Group (HOA) if they performed within 1.5SD of the norms (we applied the MCI criteria relying on the Neuropsychological Tests described below) and showed normal Instrumental Activities of Daily Living (IADL, [40]). Participants entered the early Mild Cognitive Impairment Group (eMCI) if they performed below 1.5SD from the norms on any of the tests applied but had intact IADL (see [41]). Older adults who performed below 1.5SD from the norms of any test and showed mild impairments in IADL at baseline were classified as MCI [39]. The final clinical status of MCI patients was updated in November 2018 by discussing these with the referring consultants who accessed the NHS records. Those whose records confirmed the diagnosis of dementia were grouped within the converter group (MCI converter), while those whose records still reflected the diagnosis of MCI entered the non-Converter Group (MCI non-converter).

**Fig. 2:**
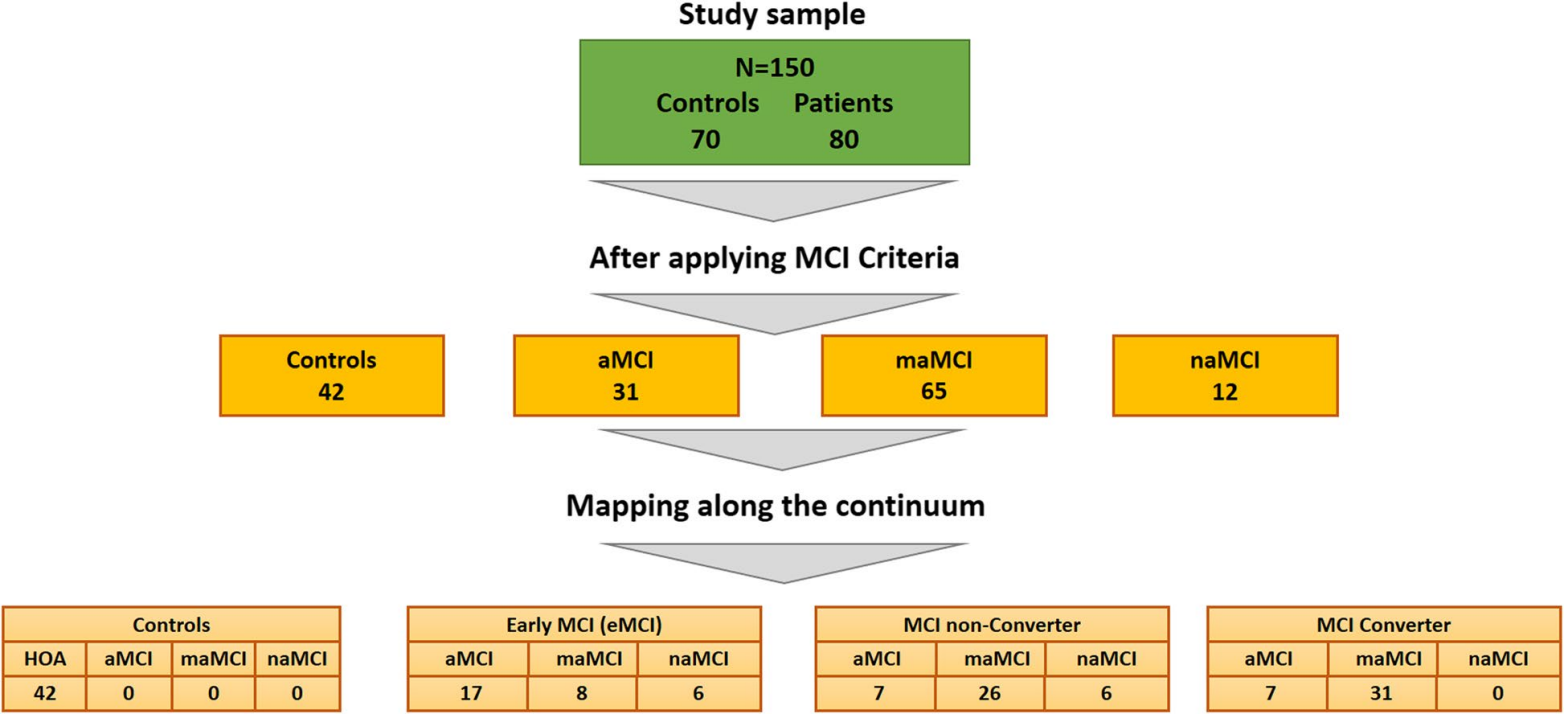
Sample collected for the present study and its classification following criteria for MCI subtypes and those used for the present study (HOA, healthy older adults; aMCI, amnestic MCI; maMCI, multi-domain amnestic MCI; naMCI, non-amnestic MCI; see text for description)

Although for the purposes of this study we did not follow the classical classification of MCI subtypes, we did apply such criteria to baseline data. Figure 2 shows the groups split after applying criteria to (1) identify MCI subtypes and (2) conform the core groups for this study. We observed a 35.2% conversion rate among MCI patients (considering eMCI and MCI) who, as Fig. 1 shows, were predominantly multi-domain amnestic MCI (maMCI). This is in line with the literature [4, 42, 43]. None of the non-amnestic MCI (naMCI) patients developed AD dementia in the course of the study, which also seems to agree with the abovementioned literature.

In order to test our first hypothesis (H1), we compared the HOA and eMCI groups (H1: Discrimination between cognitively unimpaired and older adults with very early cognitive impairment). We were interested in identifying individuals who may be displaying early signs of cognitive impairments, among the older adults who had not sought medical advice or were worried about their cognitive abilities independently of their cognitive status. We anticipated that for those displaying cognitive impairments, such impairments would be sufficiently mild as not to cause concern nor to interfere with their IADL. To test our second hypothesis (H2: Discriminate between older adults with MCI who did and did not convert to dementia in the follow-up period) we requested an updated diagnosis from the referring consultants as described above. This allowed us to retrospectively define two groups, MCI converter and MCI non-converter. The demographic, clinical, and cognitive characteristics of these groups are presented in Table 1.

**Table 1:** Descriptive statistics for the demographic variables, general cognitive and functional scales, and the neuropsychological and experimental tasks drawn from baseline assessments (*HOA* healthy older adults, *aMCI* amnestic MCI, *maMCI* multi-domain amnestic MCI, *naMCI* non-amnestic MCI; see text for description)

	HOA (***n***=42)		eMCI (***n***=31)		MCI non-converter (***n***=39)		MCI converter (***n***=38)	
	M (SD)	Range	M (SD)	Range	M (SD)	Range	M (SD)	Range
Age	73.50 (5.37)	62.00–86.00	75.71 (7.17)	61.00–90.00	75.33 (6.57)	64.00–88.00	77.08 (8.24)	58.00–97.00
Education	16.19 (4.14)	10.00–33.00	14.87 (3.52)	10.00–23.00	12.21 (3.61)	9.00–25.00	13.82 (4.20)	9.00–25.00
Total ACE-R	96.12 (3.44)	87.00–100.00	92.58 (5.61)	80.00–100.00	81.74 (7.64)	66.00–97.00	76.21 (9.45)	54.00–91.00
TOPF	63.58 (6.50)	34.00–70.00	60.16 (10.07)	32.00–69.00	49.55 (14.46)	18.00–69.00	52.23 (11.70)	31.00–69.00
IADL	7.64 (0.81)	5.00–8.00	7.33 (1.23)	5.00–8.00	6.39 (1.89)	1.00–8.00	5.93 (1.54)	3.00–8.00
HVLT delayed	9.45 (1.47)	7.00–12.00	5.65 (3.72)	0.00–12.00	3.68 (3.94)	0.00–17.00	1.76 (2.41)	0.00–9.00
HVLT total	27.26 (4.01)	17.00–36.00	22.71 (6.24)	11.00–35.00	15.45 (5.23)	6.00–26.00	13.63 (4.04)	6.00–23.00
HVLT recognition	11.43 (0.78)	9.00–12.00	9.81 (2.12)	4.00–12.00	7.94 (2.71)	0.00–12.00	7.66 (2.91)	1.00–12.00
Rey figure (copy)	34.46 (1.93)	29.00–36.00	27.87 (11.69)	0.00–36.00	28.77 (8.21)	0.00–36.00	28.06 (9.86)	0.00–36.00
Rey figure (immediate recall)	19.79 (7.67)	8.00–34.00	15.03 (9.16)	0.00–32.00	10.53 (7.61)	0.00–27.00	7.36 (6.12)	0.00–24.00
Rey figure (delayed recall)	19.50 (7.26)	7.00–34.00	14.95 (9.38)	0.00–34.00	10.49 (7.88)	0.00–26.00	5.71 (5.91)	0.00–21.00
TMT-A	43.29 (9.96)	22.00–59.00	50.97 (19.38)	25.00–103.00	66.00 (27.64)	29.00–159.00	78.35 (41.55)	35.00–252.00
TMT-B	84.95 (27.63)	34.00–163.00	107.45 (39.79)	44.00–203.00	179.53 (101.07)	72.00–547.00	196.67 (90.87)	76.00–465.00
Letter fluency (FAS)	50.81 (14.42)	18.00–81.00	49.52 (12.00)	19.00–81.00	31.57 (14.20)	14.00–67.00	34.29 (16.58)	6.00–74.00
Digit-symbol	61.64 (13.43)	36.00–83.00	54.61 (13.22)	29.00–91.00	39.74 (12.52)	14.00–62.00	34.35 (12.29)	4.00–53.00
VSTMBT (recognition)	0.75 (0.09)	0.59–0.97	0.68 (0.08)	0.53–0.78	0.64 (0.07)	0.47–0.78	0.62 (0.10)	0.41–0.81
FCSRT (free recall)	27.43 (6.17)	14.00–40.00	24.26 (7.83)	12.00–38.00	15.05 (7.90)	3.00–35.00	10.47 (6.58)	1.00–24.00

*ACE-R* Addenbrooke’s Cognitive Examination Revised, *FCSRT* Free and Cued Selective Reminding Test, *HVLT* Hopkins Verbal Learning Test, *IADL* Instrumental Activities of Daily Living, *TOPF* Test of Premorbid Functions, *TMT-A* Trials Making Test Part A and B (TMT-B), *VSTMBT* Visual Short-Term Memory Binding Test

### Assessments

A battery of neuropsychological tests was administered to all participants. The battery consisted of a combination of traditional neuropsychological tests commonly used to assess dementia [44, 45] and more novel tasks, including the VSTMBT and the FCSRT. Baseline and follow-up assessments were carried out a year apart.

### Neuropsychological assessment

The Addenbrooke’s Cognitive Examination Revised (ACE-R) was used as a Global Cognitive screening test [46]. Memory tests included the Hopkins Verbal Learning Test Immediate Total and Delayed Recall [47];) and visual memory (Rey-Osterrieth Complex Figure Immediate and Delayed Recall [48]). Assessment of attention/executive functions (TMT-A and TMT-B [49]), praxis (Rey-Osterrieth Complex Figure Copy [48]), and language/executive functions (Phonological - FAS - Fluency [50]). Speed of processing was assessed with the Digit to Symbol Substitution Test [51]. The premorbid function was assessed with TOPF [52]. We also administered the Instrumental Activities of Daily Living (IADL) Scale [40].

### Experimental tasks

The FCSRT test began with participants examining a card containing the names of objects (Grober et al., 1988). Each card showed four names, each belonging to a unique semantic category. For instance, ‘banana’ would be an example of a to-be-remembered object with the semantic cue of ‘Fruit’. Each participant learned 16 names of objects, distributed in four printed flashcards presented one at a time with four names of objects on each card. Immediate Free Recall was assessed by asking participants to retrieve the 16 names of objects spontaneously. Cued recall was subsequently assessed with the aid of the semantic cue for those items not recalled under free recall. This procedure was repeated 3 times, with a 20-s interference (counting backwards). The final Immediate Free Recall score is the sum of objects recalled from the three trials, with a minimum score of zero and a maximum of 48. The final total recall score is the sum of free recall and cued recall from all three trials, with a minimum score of 0 and a maximum of 48. We did not assess Delayed Recall in this protocol.

The VSTMB test consisted of three conditions. First, a perceptual binding task was given, as a screening test aimed at ruling out perceptual binding deficits of colour and shape [15]. Each trial began by presenting participants with two arrays of items on a computer screen (see [15] for a full description of the items’ psychophysics properties including the perceptual impact of the number of sides, colour luminance, and screen dimensions relative to foveal vision). The task was to decide if the two arrays, one on the lower half of the screen and the other on the top half, presented the same or different coloured shapes. Ten trials were included in this screening. A cut-off score of 80% was used to decide who would progress to the memory binding test [15]. All the participants who entered the study met such a criterion. We then presented the two memory conditions. The memory assessment was based on a change detection paradigm. The task comprised two conditions, Shape Only and Shape-Colour Binding. In the Shape Only condition, the study array consisted of three black shapes presented for 2000 ms. This was followed by a retention period (blank screen) for 1000 ms. Finally, a test array was presented, with three shapes in different locations to the study array. At this point, participants were required to respond ‘same’ or ‘different’. In 50% of the trials, the shapes in the study and test array were the same while in the rest of the trials, two shapes not presented at the study appeared in the test display. A similar procedure was followed in the Shape-Colour Binding condition. The to-be-remembered items were combinations of shape and colour. Participants were required to decide if the specific colour and shape combination in the test array was the same as presented in the study array (see Supplementary Figure 1 for example trials). There were 32 trials in each condition. The final score was the percentage of correct recognition. While there are currently no reported studies that have investigated the psychometric properties of the VSTMB test per se, the change detection paradigm, upon which the test is based, has been demonstrated to hold internal consistency (Logie et al., 2009). For the present analyses, we focused on the performance on the Shape-Colour Binding condition of the VSTMBT which achieved the best classification power in AD studies [15, 53, 54]. As for the FCSRT, we chose Immediate Free Recall as this proved to be the most sensitive score to detect AD [55, 56].

### Statistical analysis

To compare groups, we used tests of mean differences (t-Tests, MANOVA/MANCOVA). We also used stepwise linear regression models to investigate the individual and complementary value of the VSTMBT and the FCRST to predict group membership. To test H1 we compared HOA, eMCI and MCI non-Converter groups. To test our second hypothesis (H2) we compared eMCI, MCI non-Converter and MCI Converter groups. We also ran contrasts across groups to explore whether and to what extent the classification power of these memory tests varies as a function of the diseases continuum. The rationale was that by comparing HOA vs eMCI vs MCI non-Converter we would be able to explore the transition from normal to pathological ageing. By keeping eMCI participants separate from those who entered the MCI group (see classification criteria above) we could compare the investigated memory markers across stages where cognitive decline has had different levels of impact (i.e. from subtle without IADL impact, akin to Objectively Defined Subtle Cognitive Decline [23]) and those with overt impact on IADL. Hence, by comparing eMCI vs MCI non-Converter vs MCI Converter, we would have the opportunity to map the outcomes from these memory markers to the disease continuum and in so doing test the hypotheses set out for this study. We were also interested in the complementary value of these tests (VSTMBT and FCSRT). We defined complementary value as the ability of these tests to account for larger between-group variance (i.e. adjusted *R*^2^ from regression model) when used jointly and individually.

## Results

### General neuropsychological findings

Summaries of the descriptive and inferential statistics for the demographic variables, general cognitive and functional scales, and the neuropsychological and experimental tasks are presented in Tables 1 and 2, respectively. Of note, HOA and eMCI participants did differ on a number of neuropsychological tasks, but as anticipated, IADL were preserved. eMCI participants and MCI non-converter patients significantly differed on most neuropsychological tasks, confirming that the former group still was in the very early stages of the disease continuum. MCI non-converter and converter significantly differed from HOA on all the neuropsychological tasks. However, as MCI patients progressed along the disease continuum (i.e. MCI non-converter and converter), discrepancies on neuropsychological scores became less apparent. Although this study focused on novel neuropsychological tests, some well-established standardised tests showed excellent abilities to discriminate between individuals in the early stages (HVLT in HOA vs eMCI). Regarding the experimental tasks, the ability of FCSRT and VSTMT to predict group membership showed differences throughout the disease continuum, with opposite patterns of sensitivity at its extremes (preclinical: VSTMBT > FCSRT, advanced prodromal: FCSRT > VSTMBT), and varying levels of complementary status throughout its intermediate stages (see Fig. 2).

**Table 2:** Inferential statistics with the demographic variables, general cognitive and functional scales, and the neuropsychological and experimental tasks (*HOA* healthy older adults, *aMCI* amnestic MCI, *maMCI* multi-domain amnestic MCI, *naMCI* non-amnestic MCI; see text for description)

	HOA vs eMCI		HOA vs non-converter		HOA vs converter		eMCI vs non-converter		eMCI vs converter		Non-converter vs converter	
	(***t***)	***p***-value	(***t***)	***p***-value	(***t***)	***p***-value	(***t***)	***p***-value	(***t***)	***p***-value	(***t***)	***p***-value
Age	− 1.51	0.140	− 1.38	0.170	− 2.28	0.030	0.23	0.820	− 0.73	0.470	− 1.03	0.310
Education	1.43	0.160	4.60	<0.001	2.54	0.01	3.10	<0.001	1.11	0.270	− 1.80	0.080
Total ACE-R	3.11	<0.001	10.77	<0.001	12.27	<0.001	6.60	<0.001	8.92	<0.001	2.83	0.010
TOPF	1.64	0.110	5.48	<0.001	4.85	<0.001	3.58	<0.001	2.86	0.010	− 0.83	0.410
IADL	0.95	0.350	3.40	<0.001	5.07	<0.001	1.76	0.09	3.03	<0.001	1.04	0.300
HVLT delayed	5.40	<0.001	8.42	<0.001	17.02	<0.001	2.11	0.04	5.02	<0.001	2.53	0.010
HVLT total	3.56	<0.001	11.24	<0.001	15.13	<0.001	5.26	<0.001	7.00	<0.001	1.69	0.090
HVLT recognition	4.04	<0.001	7.25	<0.001	7.73	<0.001	3.07	<0.001	3.44	<0.001	0.43	0.670
Rey figure (copy)	3.11	<0.001	4.22	<0.001	3.84	<0.001	− 0.38	0.71	− 0.07	0.940	0.34	0.730
Rey figure (immediate recall)	2.40	0.020	5.42	<0.001	7.94	<0.001	2.25	0.03	3.98	<0.001	1.99	0.050
Rey figure (delayed recall)	2.31	0.020	5.19	<0.001	9.07	<0.001	2.09	0.04	4.68	<0.001	2.89	0.010
TMT-A	− 2.02	0.050	− 4.79	<0.001	− 4.81	<0.001	− 2.56	0.01	− 3.45	<0.001	− 1.50	0.140
TMT-B	− 2.85	0.010	− 5.58	<0.001	− 6.82	<0.001	− 4.03	<0.001	− 5.14	<0.001	− 0.75	0.460
Letter fluency (FAS)	0.41	0.690	5.96	<0.001	4.64	<0.001	5.56	<0.001	4.20	<0.001	− 0.75	0.460
Digit-symbol	2.22	0.030	7.52	<0.001	9.14	<0.001	4.79	<0.001	6.40	<0.001	1.84	0.070
VSTMBT (recognition)	3.49	<0.001	5.78	<0.001	5.92	<0.001	2.12	0.040	2.71	0.010	1.11	0.270
FCSRT (free recall)	1.93	0.060	7.89	<0.001	11.73	<0.001	4.86	<0.001	7.83	<0.001	2.71	0.010

*ACE-R* Addenbrooke’s Cognitive Examination Revised, *FCSRT* Free and Cued Selective Reminding Test, *HVLT* Hopkins Verbal Learning Test, *IADL* Instrumental Activities of Daily Living, *TOPF* Test of Premorbid Functions, *TMT-A* Trials Making Test Part A and B (TMT-B), *VSTMBT* Visual Short-Term Memory Binding Test

### Transition from normal to pathological ageing

To predict group membership in the very early stages of cognitive decline, we focused on data (FSCRT and VSTMB) from HOA, eMCI and MCI non-converter. To test H1, we first relied on tests of mean differences (MANOVA/MANCOVA or t-Tests) and later used stepwise linear regression models (see Table 3). Both tests displayed excellent abilities to discriminate between HOA, eMCI and MCI non-converter (HOA > eMCI/MCI non-converter). The VSTMB outperformed the FCSRT only in the preclinical stages (HOA > eMCI).

**Table 3:** Results from MANOVA/MANCOVA and Regression analyses investigating the individual and complementary value of the VSTMBT and FCSRT (*HOA* healthy older adults, *aMCI* amnestic MCI, *maMCI* multi-domain amnestic MCI, *naMCI* non-amnestic MCI; see text for description)

	**HOA vs eMCI**				**HOA vs non-converter (!)**				**HOA vs converter ($!)**			
	**MANOVA**		**Regression (14%,*p*=0.001)**		**MANCOVA**		**Regression (57%,*p*<0.001)**		**MANCOVA**		**Regression (65%,*p*<0.001)**	
	***F***	***p***	***t***	***p***	***F***	***P***	***t***	***P***	***F***	***p***	***t***	***p***
**VSTMB (% recognition)**	12.19	0.001	− 3.49	0.001	20.26	<0.001	− 5.32	<0.001	22.20	<0.001	− **2.98**	**<0.001**
**FCSRT (free recall)**	4.39	0.040	− **1.86**	**0.067**	41.54	<0.001	− 3.11	0.003	94.20	<0.001	− 8.39	0.004
	**eMCI vs non-converter (!)**				**eMCI vs converter**				**Non-converter vs converter**			
	**MANCOVA**		**Regression (30.8%,*p*<0.001)**		**MANOVA**		**Regression (44.4%,*p*<0.001)**		**MANOVA**		**Regression (6.1%,*p*=0.025)**	
	***F***	***p***	***t***	***p***	***F***	***P***	***t***	***P***	***F***	***p***	***t***	***p***
**VSTMB (% recognition)**	2.27	0.137	− **0.93**	**0.354**	7.13	0.010	− **0.84**	**0.403**	1.20	0.277	− **0.45**	**0.651**
**FCSRT (free recall)**	16.20	<0.001	− 4.02	<0.001	49.78	<0.001	− 7.06	<0.001	5.24	0.025	− 2.29	0.025

$: adjusting for age; !: adjusting for education; % = percentage of variance accounted for by the model which comprises both predictors. Bold font: excluded from model. MANCOVA were conducted when either age or education was significant; otherwise, MANOVA models were used; *t*-values correspond to separate hypothesis tests for each predictor variable (with the corresponding *p*-value) (see Supplementary Table 1 for full report on regression models)

### Exploring the prodromal stages

To explore the more advanced prodromal stages of the disease, we focused on data from eMCI, MCI non-converter and MCI converter. The same analytical approach was followed (see Table 3). Relative to the FCSRT, the VSTMB proved less effective in discriminating eMCI from MCI non-Converter, eMCI from MCI Converter, and MCI non-Converter from MCI Converter. In fact, regression models showed that the VSTMB was excluded as a predictor from all the above contrasts, which only retained the FCSRT. The MANOVA/MANCOVA analyses confirmed that such a limited predictive value of the VSTMBT relative to the FCSRT is explained by its reduced ability to differentiate between groups as soon as patients moved into the prodromal stages of the disease, the point at which the FCSRT becomes more sensitive. These findings too support our H1 and H2.

### Exploring the individual and complementary value of the VSTMBT and FCSRT

**Fig. 3:**
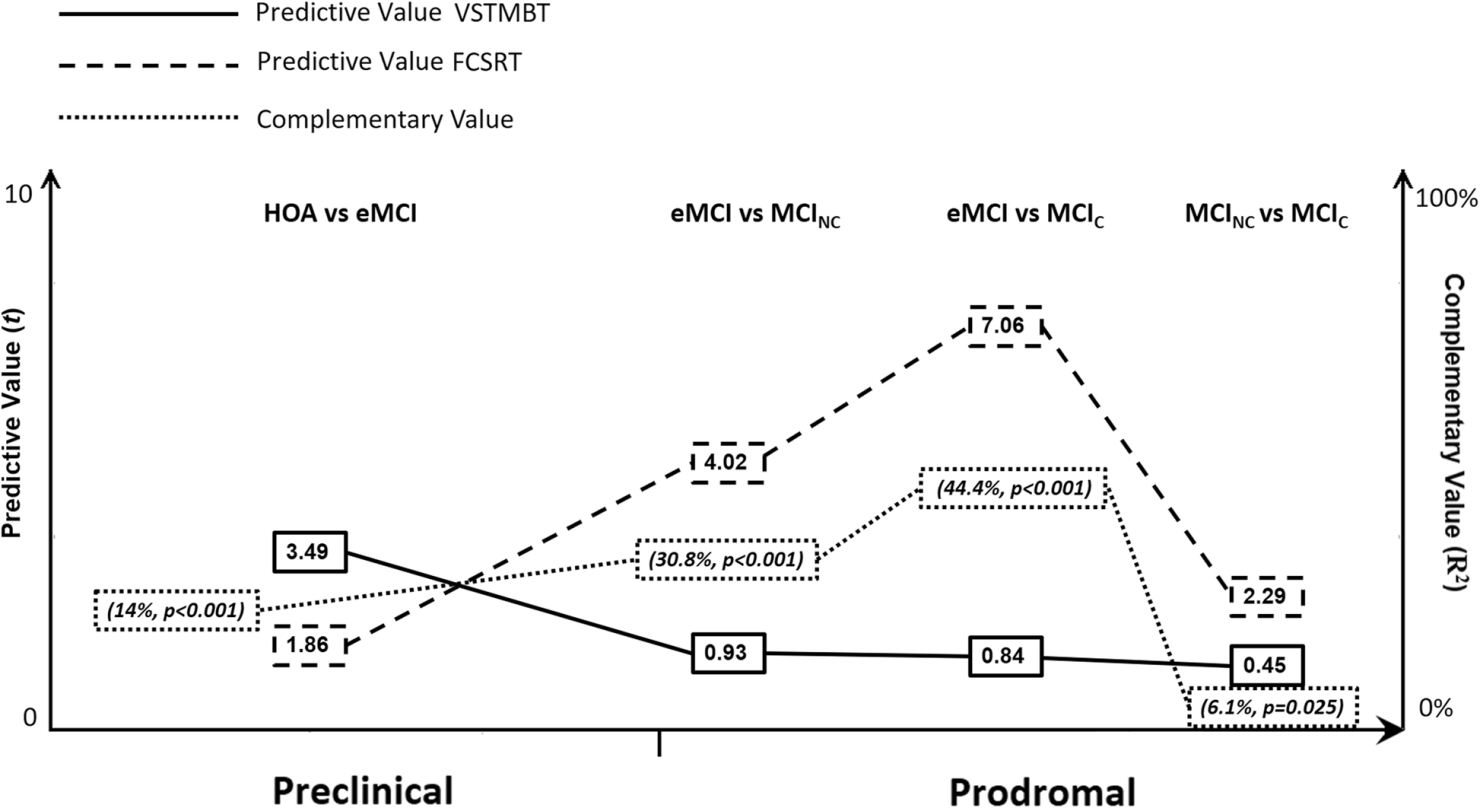
Diagram representing the individual and complementary value of the VSTMBT and FCSRT for the prediction of group membership along the continuum from the pre-symptomatic to prodromal stages of AD clinical syndrome

As reported above, the VSTMBT proved a good predictor of group membership in the early preclinical stages. As soon as the levels of cognitive impairment met criteria for the prodromal stages of the continuum, the FCSRT outperformed the VSTMBT. As Table 3 and Fig. 3 show, the complementary value of these tasks varied as the level of cognitive impairment progressed. It was low at the extremes of the stages of the continuum here explored and higher in the medium stages. The individual and combined predictive value of both tests to discriminate between stages closer to dementia (MCI non-converter and MCI converter) was rather low.

## Discussion

The present longitudinal study was set out to investigate the hypotheses that two memory markers for AD recently recommended by consensus [10] would differently predict dementia throughout its continuum. Based on previous evidence we predicted that the VSTMBT would be able to discriminate between older adults who are asymptomatic and those who are in the very early stages of cognitive decline more effectively than the FCSRT (H1). However, the FCSRT would discriminate between older adults in the prodromal stages (MCI) who later convert versus those who do not convert to dementia more accurately than the VSTMBT (H2). Our results supported both hypotheses and have some implications for our understanding of neuropsychological assessment to track the transition from normal to pathological ageing and to monitor progression throughout the prodromal stages towards conversion to dementia.

Before discussing these implications, it is worth considering some observations drawn from the background neuropsychological assessment. HOA and eMCI participants differed on a number of neuropsychological tasks, yet eMCI participants were not seeking professional help. As patients with MCI progressed along the disease continuum (i.e. MCI non-Converter and Converter), discrepancies in the neuropsychological scores decreased. Hence, standard neuropsychological tests used in our study appear to be effective for detecting impairments but less so for differentiating risk phenotypes. These shortcomings of off-the-shelf neuropsychological tests have been acknowledged previously [8, 57–60] and called for new tests to better phenotype dementia and detect risk profiles [8, 57]. Notwithstanding such limitations, the ability of some neuropsychological tests used in our assessment battery to detect very early cognitive impairments, particularly of memory, is also worth highlighting.

The HVLT revealed significant memory differences along the disease continuum, particularly between groups informing the very early stages. Lonie et al. [61] had previously demonstrated that the Delayed Recall component of the HVLT can discriminate between MCI converters and non-converters over a 4-year follow-up period as accurately as the Visuospatial Paired Associates (PAL) Task from CANTAB [62]. Gustavson et al. [63] recently reported that the California Verbal Learning Test, which assesses constructs similar to those tested by the HVLT, is an early indicator of MCI risk at an age when few individuals are likely to have yet become biomarker positive. Regarding the experimental tasks, the FCSRT and VSTMT showed differential abilities to predict group membership along the disease continuum. Opposite patterns of sensitivity were observed at the extreme ends of the continuum here explored (preclinical: VSTMBT > FCSRT; advanced prodromal: FCSRT > VSTMBT), and varying levels of complementary status throughout its intermediate stages. These findings lend support to the two hypotheses investigated in this study and suggest that these recently recommended tests [10, 14, 53, 64] shall form part of new memory toolkits to assess and monitor AD.

To investigate the individual and complementary values of the two experimental tasks in informing about the transition from normal to pathological ageing we focused on data from HOA, eMCI and MCI non-Converter. We predicted that the VSTMBT should discriminate well in the earlier stages because it would be able to detect gradually increasing levels of impairments whereas the function assessed by the FCSRT would still be preserved. Didic et al. [12] suggested that AD affects medial temporal lobe (MTL) structures known to support different memory functions in a graded manner (Fig. 1). Within the MTL, the disease first goes through a subhippocampal stage (Braak and Braak’s stages I and II, which correspond to the asymptomatic stages) selectively impairing regions of the anterior MTL network (i.e. perirhinal and lateral entorhinal cortex, the anterior hippocampus, and the temporo-polar cortex). Damage to these regions impairs context-free memory [65, 66]. As the disease progresses to the limbic stage (i.e. Braak and Braak stages III and IV, which correspond to the mild cognitive impairment stage (MCI)), pathology spreads to the posterior MTL network (i.e. parahippocampal cortex, posterior hippocampus, and posterior cingulate) which plays a critical role in context-rich memory. Hence, this model predicts that memory functions such as those assessed by the VSTMBT would be affected earlier than those assessed by selective reminding paradigms such as the FCSRT and the MCT.

Dissociations of these forms of binding in populations with or at risk of AD have been observed earlier. Studies carried out in preclinical samples of carriers of mutations that inevitably lead to familial AD (i.e. E280A-PSEN1 [54, 67]) reported VSTMB deficits in asymptomatic carriers who were about 10 years younger than the average age of onset of dementia in this familial variant. However, using the MCT to assess members of the same kindred, Romero –Vanegas et al. [68] found impairments only when carriers were in the MCI stages. Using another context-rich memory test memory test, the Paired Associates Learning of WMS [69]), Parra et al. [54] reported that the VSTMBT significantly outperformed it when discriminating between asymptomatic carriers and non-carrier controls. Similar results were reported by Koppara et al. [70] in patients with subjective cognitive decline who presented with VSTMB impairments within an otherwise normal neuropsychological profile. We further observed that in confirmed cases of AD, both tests achieved excellent levels of classification at the individual level, even if the VSTMBT outperformed the FCSRT [53].

More recent studies that combined VSTMB tests with biomarker assessments (i.e. PET), have confirmed that such ability is affected by the very early stages of AD. For instance, Norton et al. [71] reported correlations between performance on the VSTMBT and amyloid deposits in asymptomatic carriers of the mutation E280A-PSEN1. Interestingly, when the disease progressed to the symptomatic stages, such correlations were no longer statistically reliable, likely due to a profound impairment in VSTMB (performance close to floor). The authors suggested that VSTMB impairments may effectively predict dementia in those affected by AD in the preclinical stages. The same research group [72] recently reported correlations between performance on context-rich tests (Latin American Spanish version of the Face-Name Associative Memory Exam). Significant correlations were not observed when data from only asymptomatic carriers entered the statistical models. However, when data from asymptomatic and symptomatic carriers were lumped together, correlations between memory scores and amyloid deposits reached significance, suggesting that when it comes to context-rich memory tests, such an association becomes apparent in rather advanced pathological stages. Relying on the recently proposed biomarker framework [6], Papp et al. [73] reported evidence of impairment in the binding component of the MCT (i.e. cued recall) only between participants in Stage 0 (Aβ−/ND−) and Stage 2 (Aβ+/ND+), but not between those in Stage 0 and Stage 1 (Aβ+/ND−). Cecchini et al. [74] recently reported that deficits in VSTMB are detectable in individuals with brain amyloid deposition in the absence of overt neurodegeneration (N aspect of the A/T/N framework, [6]) in the AD continuum. Taken together studies which included the VSTMBT, selective reminding paradigms, and AD biomarkers lend support to Didic et al.’s [12] hypothetical model.

In fact, accrued evidence using the FCSRT suggests that mapping memory decline along the AD continuum is now possible. The test has unveiled memory decline during subclinical Aβ levels [75] and has demonstrated to be able to predict incident MCI [76–78]. Variables drawn from this test have proved informative of the stages of such a continuum [79, 80]. For instance, a decline in free recall, which is linked to retrieval impairments, tends to inform about stages where either Aβ [77] or mild tauopathy [81] become detectable in still asymptomatic individuals. However, a decline of total recall, which is linked to retrieval and storage impairments (demonstrated by the inability to benefit from cuing), seems to reflect the symptomatic stages (Aβ, tau, and neurodegeneration, [80]). As it is encouraging, this evidence also triggers several questions.

Are current approaches to staging the AD continuum appropriate? The recently proposed framework is aimed at detecting neuropathological signatures using biomarkers [5, 6]. Should these efforts prove fruitful, strategies then focus on identifying the cognitive and functional decline that ensues (e.g. [11, 82]). A growing number of studies are reporting cognitive deficits in subthreshold [33, 63, 75, 83, 84] and subclinical [71, 73, 79] stages of the disease continuum. The former evidence comes from studies using Aβ markers in the brain, CSF or blood [5, 6]. The latter is commonly documented using the Clinical Dementia Rating Scale (CDR=0) [85]. There is consensus that current biomarkers for AD lack specificity [86, 87]. Moreover, the CDR does not allow staging the preclinical/predementia stages of AD, which have become the most investigated in recent years [41, 88, 89]. Therefore, we need to continue refining our understanding and tools to better map cognition along the AD continuum, particularly in its preclinical stages.

Are we mapping promising memory markers for AD onto the correct neural correlates? Based on current understanding [12, 20, 90], tests that tap into the function of the hippocampus are not good candidates to detect the pre-symptomatic stages of AD (i.e. transentorhinal stages, see Fig. 1). So, what brain regions are the memory binding tests investigated here really taxing? Neuroimaging studies have consistently confirmed that the sensitivity of the FCSRT lies on its ability to index the function of the hippocampus [80, 91]. Of note, VSTMB functions can be carried out without functional hippocampi [92–94]. The evidence that VSTMB deficits are associated to increase Aβ prior to tau pathology [71] and neurodegeneration [74] suits the available neuroanatomical evidence. However, if the FCSRT is informing about the hippocampal stages of AD, the association of such deficits to Aβ deposits (i.e. Stage of Objective Memory Impairment 1, [80]) prior to tau pathology becomes more challenging to interpret. Parra [22] recently suggested the need to zoom out if we are to unveil more promising neural correlates of memory functions sensitive to AD. Future efforts will be needed to continue mapping these memory markers onto the continuum of neuropathological events that lead to AD dementia.

The variability of results across the studies discussed above could also reflect task-related artefacts rather than meaningful cognitive decline. For instance, over the last few years, the FCSRT has undergone substantial revisions to improve its construct and cultural validity. Buschke [26] revised the task to improve the binding construct via the memory capacity test (MCT). In fact, Papp et al. [73] recently showed that the MCT version holds sensitivity for the preclinical amyloidosis seen in Stage 1 (free recall) and the amyloidosis and neurodegeneration seen in Stage 2 (free and cued recall). The task has been devised in both “word” and “picture” formats with the latter yielding better outcomes [95]. Due to the superiority effect of pictures over words and practice at cued recall in the study phase before the test phase [96], scores on the two versions are quite different (an 8-point difference in FR and a 4-point difference in TR [95]). This can explain why the picture version of the FCSRT has yielded better results in illiterate populations [97].

The VSTMBT too has undergone scrutiny. For instance, an earlier version of the test followed titration procedures [15, 54] which allowed confirming the specificity of such a deficit but would be too challenging for implementation in clinical settings [53]. Parra et al. [31] later reported the different task settings that may suit different research aims. For instance, the 2-item version was suggested as the most suitable for the symptomatic stages whereas the 3-item version would achieve the best sensitivity in pre-symptomatic stages. Such proposals have been neither extensively explored nor confirmed. Therefore, as suggested by the Joint Program for Neurodegenerative Diseases Working Group [10], the two tests have proved informative of preclinical AD but their complementary value needs to be further investigated in order to address the above knowledge gaps (e.g. [98]).

In the current study, we demonstrated that both tests hold excellent abilities to discriminate between HOA, eMCI and MCI non-Converter (HOA > eMCI/MCI non-Converter). The VSTMB outperformed the FCSRT only in the preclinical stages (HOA > eMCI). As the disease progressed, (i.e. eMCI/MCI non-Converter) performance on the VSTMBT became less differentiated between groups, whereas that on the FCSRT continued to effectively discriminate between them. These findings, although encouraging, raise a number of concerns for promising neuropsychological assessments aimed at the preclinical stages of AD. Logie et al. [14] suggested that a good memory marker for AD should avoid very low-performance levels when the symptoms become severe. Regarding the VSTMBT, which relies on the Change Detection Paradigm, chance levels are set at 50%. This is a constraint of the method. To overcome it, Parra et al. [31] suggested strategies such as titrating the task difficulty (i.e. memory load, see above and also [54, 67, 70]). In the present study, we chose to use one set size (i.e. 3) for the sake of comparability of findings along the disease continuum, particularly considering our aim of anticipating the MCI stages [9, 23, 63, 83, 84].

The literature supporting the validity of the FCSRT to predict dementia in longitudinal cohorts of MCI patients has grown significantly over the last few years (e.g. [28, 77, 79, 80, 99–103]). This is the first report on the use of the VSTMBT in such longitudinal cohorts. Our results support the notion that the neuropsychological assessment of AD, in its new conceptualization (i.e. a continuum of clinical and pathological stages), ought to abandon the one-size-fits-all approach. Assessment protocols aimed at investigating AD-related disorders (i.e. detection, prediction) need to consider the evidence presented here. Belleville et al. [104] acknowledged that a cognitive toolkit intended to identify AD at the pre-dementia stage would need tasks that are early indicators and others that might suggest imminent progression.

The fact that the VSTMBT detects AD-related changes early (see [54, 67, 70]) and then performance drops to near or chance levels (see [71]) has pros and cons. The positive aspect of this is that we have long-needed tests that can detect the very early stages of the disease process, preferably, when people are unaware of or are very little concerned about any cognitive or functional impairment. We have learned that at this stage, the VSTMBT is taxing the early accumulation of amyloid in at-risk individuals even before tau deposits or neurodegeneration become apparent [33, 71, 74]. Such a test would be an ideal tool for clinical trials aiming at dementia prevention as they could enhance recruitment strategies by selecting who will likely meet inclusion criteria (e.g. Aβ+).

One final aspect concerns our control participants. Most participants who were allocated to the eMCI group entered the study as self-referred healthy volunteers (see Fig. 2). Relative to those who met criteria for HOA, eMCI participants displayed significant differences on various standard neuropsychological assessments. This is striking, as these individuals, at the time of the study, had not sought help and a few were only mildly concerned about their cognitive abilities. There is consensus that in the new context of AD research and clinical practice (i.e. following the biological definition of AD), deciding who is a control individual is proving as challenging as deciding who is in the early stages of the disease [32]. There are two issues worth considering here. First, the source of these control volunteers and second, awareness of and stigmas against early symptoms of dementia. Volunteers entering as controls were recruited from the Psychology Volunteer Panel at the University of Edinburgh o were relatives of patients with dementia. In the case of the former source, there is awareness about the impact that such selective samples could have on the interpretation of data [105]. Older adults involved in such panels (1) regularly support research and (2) are often highly educated, thus representing a rather biassed sub-sample of the relevant population. Importantly, they frequently undergo cognitive testing, which grants them additional cognitive reserves and resilience [29, 106, 107]. Therefore, it is not entirely surprising that these older adults overlook or underestimate the level of decline in cognitive abilities here identified. Although volunteering has been considered a protective action against cognitive decline [106], managers of volunteer panels need to be aware of these risks. In the case of the latter source of recruitment (i.e. relatives of patients with dementia), there is evidence that the burden posed by the patients’ level of cognitive and behavioural problems causes caregiver stress, which in turn leads to impaired cognitive functioning [108, 109]. Therefore, volunteer panels and dyads of dementia patients, two common sources of recruitment in ageing and dementia studies, will need revised approaches if we are going to progress in the new dementia research context with more confidence and reliability. The second issue, awareness of and stigmas against early symptoms of dementia, is also relevant [110] and suggests that more work is needed to continue raising awareness about the fact that ageing is not a disease [111] and that seeking help early is the best approach to mitigate the dramatic impact that departures from its normal trajectory will carry.

### Limitations

There are some limitations that need to be considered when interpreting the findings here reported. The first one is the rather small sample size. However, as shown by our inferential statistics, effect sizes were rather large for the hypotheses tested. Moreover, both experimental tests used in this study have demonstrated to hold informative value to identify individual patients and not just during group comparisons. For instance, the VSTMBT test had shown sensitivity and specificity value of over 77% in completely asymptomatic individuals [54] and of 100% for patients with dementia (see Della Sala et al. [53] who reported an area under the curve of 96% for the FCSRT). Nevertheless, efforts will be needed to expand such samples within disease stages and along the continuum, and such efforts are already ongoing [112].

Another limitation is the nature of the control participants who entered this study. This is not a representative sample. Even if unpaired cognitively, it is still possible that some of these older adults were already accumulating disease pathology (see [33]). Together with the report by Parra et al. [31], this evidence suggests that some of those who entered our HOA group may still be classified as not healthy controls if the approach recommended by Bos et al. [32] is followed. This limitation is shared by many studies in the field and urgent strategies will be necessary to address this important caveat. Although we monitored our participants yearly, we did not receive confirmation of the precise date the dementia diagnosis was given but rather the clinical status at the end of the study (see Methods for more details). Time to dementia onset is an important variable for models aimed at investigating the predictive value of assessment tools. Future analysis involving longitudinal assessments with the VSTMBT should pursue such information. One final limitation of this study is that we did not have biomarkers evidence to assess the biological status of our MCI patients and hence we choose to adhere to the definition of Alzheimer’s clinical syndrome as recently recommended [1, 5, 6].

## Conclusions

In the current longitudinal study, we have demonstrated that neuropsychological assessments for AD shall move away from the notion of one-size-fits-all. A memory toolkit for AD needs to be considered which contains tools that are early indicators and others that might suggest imminent progression. This study, the first one reporting on the use of the VSTMBT in the longitudinal assessment of MCI, suggests that the VSTMBT may provide an early indicator for such a toolkit while the FCSRT seems to be an excellent tool to assess imminent progression.

## Supplementary Information

**Additional file 1: Supplementary Table 1.** Results from the Regression Analyses.

**Additional file 2: Supplementary Figure 1.** Example trial of the Perceptual Binding Task used for screening purposes (A) and both conditions of the VSTMB task (B). See text in the manuscript (Method) for a full description of these tasks.

## Data Availability

This is the first publication emerging from this longitudinal study. The team is currently professing data to generate further publication. The data used to prepare this manuscript can be available on request.

## Abbreviations

A/T/N: Amyloid Tau Neurodegeneration Framework
ACE-R: Addenbrooke’s Cognitive Examination Revised
AD: Alzheimer’s disease
Aβ: Amyloid β
eMCI: Early Mild Cognitive Impairment Group
FCSRT: Free and Cued Selective Reminding Test
GDS: Geriatric Depression Scale
HOA: Healthy Older Adults Group
HVLT: Hopkins Verbal Learning Test
IADL: Instrumental Activities of Daily Living
maMCI: Multi-domain amnestic MCI
MANOVA/MANCOVA: Multivariate Analysis of Variance/Covariance
MCI converter: MCI patients who developed dementia in the follow-up period
MCI non-converter: MCI patients who did not develop dementia in the follow-up period
MCI: Mild cognitive impairment stage
MCT: Memory capacity test
MTL: Medial temporal lobe
ND: Neurodegeneration
NDN: Neuroprogressive and Dementia Network
NHS: National Health Services
SDCRN: Scottish Dementia Clinical Research Network interest register
SRT: Selective Reminding Tests
TMT-A and TMT-B: Trail Making Test Parts A and B
TOPF: Test of Premorbid Functions
VSTMB: Visual Short-Term Memory Binding
VSTMBT: Visual Short-Term Memory Binding Test
