# Supplementary material for: Memory markers in the continuum of the Alzheimer’s clinical syndrome
Source: Alzheimers Res Ther. 2022 Sep 30;14:142. doi: 10.1186/s13195-022-01082-9 (PMC9526252; doi:10.1186/s13195-022-01082-9)
Supplement: Supplementary file 1 — Additional file 1: Supplementary Table 1. Results from the Regression Analyses. [file 13195_2022_1082_MOESM1_ESM.docx]

**Memory markers in the continuum of the Alzheimer’s clinical syndrome**

**Mario A Parra, Clara Calia, Vivek Pattan, Sergio Della Sala**

**Supplementary Material**

**Supplementary Table 1.** Results from the Regression Analyses.

| **Model Contrasts** | **Model Outcome** | **ANOVA** | | **Testing Predictors** | | | |
| --- | --- | --- | --- | --- | --- | --- | --- |
|  |  |  |  | **VSTMB (% Recognition)** | | **FCSRT (Free Recall)** | |
|  | **Adjusted R^2^** | ***F*** | ***p*** | ***t*-test** | ***p*** | ***t*-test** | ***p*** |
| HOA vs eMCI | 0.138 | 12.19 | <0.001 | -3.49 | <0.001 | **-1.86** | **<0.001** |
| HOA vs Non-Converter | 0.569 | 33.61 | <0.001 | -5.32 | <0.001 | -3.11 | 0.003 |
| HOA vs Converter | 0.653 | 68.85 | <0.001 | **-2.98** | **0.004** | -8.39 | <0.001 |
| eMCI vs Non-Converter | 0.308 | 15.01 | <0.001 | **-0.93** | **0.354** | -4.02 | <0.001 |
| eMCI vs Converter | 0.444 | 49.78 | <0.001 | **-0.84** | **0.403** | -7.06 | <0.001 |
| Non-Converter vs Converter | 0.061 | 5.24 | 0.025 | **-0.45** | **0.651** | -2.29 | 0.025 |

Red/Bold font = excluded from the model
