# Supplementary figures and images for: Memory markers in the continuum of the Alzheimer’s clinical syndrome
Source: Alzheimers Res Ther. 2022 Sep 30;14:142. doi: 10.1186/s13195-022-01082-9 (PMC9526252; doi:10.1186/s13195-022-01082-9)

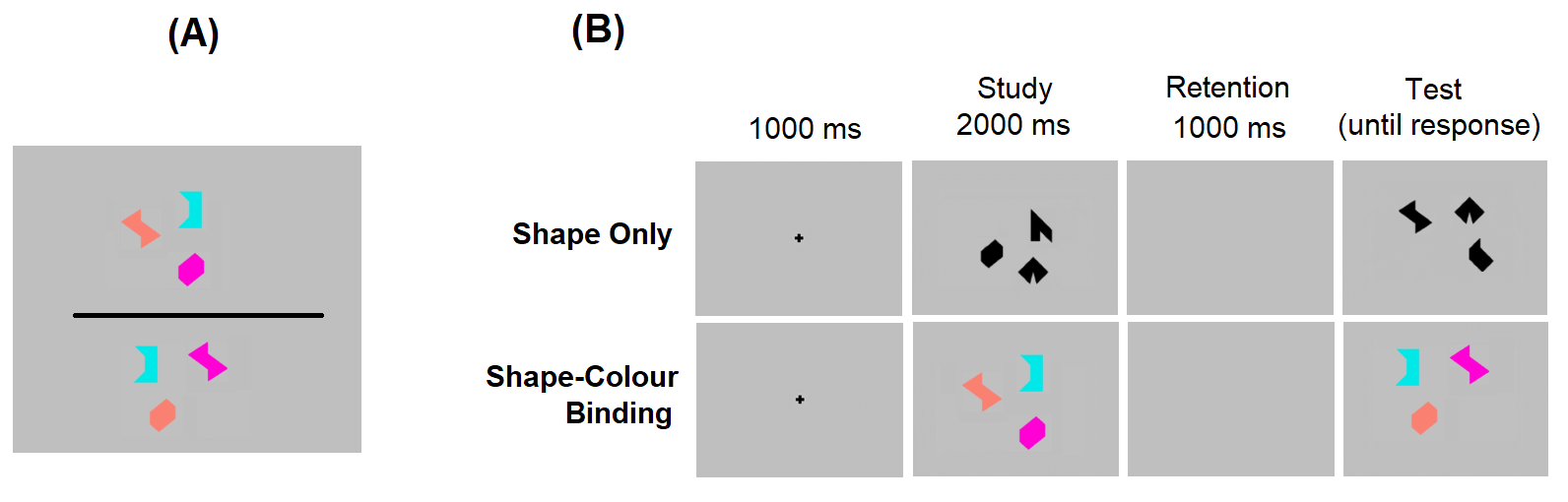

Supplement: Supplementary file 2 — Additional file 2: Supplementary Figure 1. Example trial of the Perceptual Binding Task used for screening purposes (A) and both conditions of the VSTMB task (B). See text in the manuscript (Method) for a full description of these tasks. [file 13195_2022_1082_MOESM2_ESM.tif]
